# Graves’ Disease in the Elderly: A Case of Fluctuating Hyperthyroidism and Hypothyroidism Resulting in Challenging Management

**DOI:** 10.7759/cureus.94255

**Published:** 2025-10-09

**Authors:** Charlotte Fernando, Charlotte Soan, Lakshmi Sankaran, Meenakshi Parsad

**Affiliations:** 1 Diabetes and Endocrinology, Hampshire Hospitals NHS Foundation Trust, Winchester, GBR

**Keywords:** antithyroid drugs, atypical presentation of hyperthyroidism, drug-induced hypothyroidism, elderly population, graves' disease, hyperthyroidism, hypothyroidism cardiovascular manifestations

## Abstract

A woman in her late 80s was diagnosed with Graves’ disease (GD) following routine blood tests, which showed a thyroid-stimulating hormone of <0.005 mU/L, free thyroxine of 39 pmol/L, and TSH receptor antibodies of 9.8 IU/L. She denied adrenergic symptoms. Thyroid ultrasound revealed a 37 mm benign nodule in the left lobe. She had known atrial fibrillation and heart failure, as well as a history of toxic nodular goiter treated with partial thyroidectomy 50 years earlier. On examination, she presented with exophthalmos and pretibial myxedema, initially treated as cellulitis. She was started on carbimazole 15 mg; however, thyroid function tests were not monitored. Five months later, she was admitted to the hospital with symptomatic hypothyroidism and persistent bradycardia. Carbimazole was discontinued, and levothyroxine was initiated. Over the following four months, she was admitted three more times with alternating hypothyroidism and hyperthyroidism, accompanied by worsening cardiac complications. This case highlights the challenges of recognizing and managing GD and its complications in the elderly.

## Introduction

Graves’ disease (GD) is an autoimmune condition characterized by the presence of autoantibodies against the thyroid-stimulating hormone (TSH) receptor (TSH-R), leading to unregulated production and secretion of thyroid hormones. GD has an annual incidence of 20-50 cases per 100,000 people [1], with female patients affected 10 times more frequently than males [2]. The peak incidence occurs between 30 and 50 years of age, while in patients over 60, a higher proportion of cases are due to toxic nodular goiter [2,3]. Symptoms typically associated with GD include heat intolerance, tremor, palpitations, and weight loss, although extrathyroidal manifestations such as Graves’ orbitopathy may also be observed. Diagnosis is confirmed through biochemical investigations, including thyroid function tests (TFTs) and thyroid receptor antibodies (TRAb). Treatment options include antithyroid drugs (ATDs) such as carbimazole, radioactive iodine (RAI), and thyroidectomy in selected cases.

This report describes the case of an elderly woman diagnosed with GD in the absence of the adrenergic symptoms typically associated with the disease. Her thyroid status has since fluctuated between hypothyroidism and hyperthyroidism as a result of overtreatment with alternating carbimazole and levothyroxine therapy, due to challenges with dose titration. She has experienced multiple systemic complications, either attributable to or exacerbated by thyroid dysfunction, most notably decompensated heart failure and atrial fibrillation (AF) with persistent bradycardia. In conjunction with symptomatic hypothyroidism secondary to carbimazole, these cardiac complications have led to multiple hospital admissions, which, given her frailty, have affected her functional baseline. Owing to her frailty and comorbidities, other treatment options for GD have been limited.

This case highlights the challenges of recognizing GD in the elderly population, where symptoms may be more subtle than in younger patients or attributed to other age-related conditions. In addition, it underscores the difficulties clinicians may encounter in the medical management of GD in older adults, as well as the complications that can arise if thyroid dysfunction is not adequately corrected.

## Case presentation

Our patient of interest, a woman in her late 80s, underwent blood tests in primary care a year ago for the investigation of anemia (Table 1). TFTs, included as part of the panel, showed a TSH <0.005 mU/L and free thyroxine (FT4) 39 pmol/L. TRAb were subsequently positive at 9.8 IU/L, confirming a diagnosis of GD. Although the patient initially reported no symptoms, it was later noted that she had lost 12% of her body weight in the preceding months and had experienced dysphagia and looser stools. A CT of the chest, abdomen, and pelvis, along with esophagogastroduodenoscopy and CT colon, revealed no evidence of malignancy.

**Table 1:** Laboratory investigations performed in primary care as part of the ongoing evaluation of anemia of unclear etiology Following the TSH and FT4 results, TRAb testing was requested on the same specimen. A diagnosis of GD was confirmed after a positive TRAb result. ALT, alanine aminotransferase; BUN, blood urea nitrogen; CKD-EPI, Chronic Kidney Disease Epidemiology Collaboration; eGFR, estimated glomerular filtration rate; FT4, free thyroxine; GD, Graves’ disease; MCV, mean corpuscular volume; TRAb, thyroid receptor antibodies; TSH, thyroid-stimulating hormone

Test	Lab value	Reference value
TSH	<0.005	0.27-4.2 mU/L
FT4	39	11-22 pmol/L
TRAb	9.8	0-0.4 IU/L
Hemoglobin	115	120-160 g/L
MCV	82	76-103 fL
White blood cell count	7.1	4-11 × 10⁹/L
Platelet count	259	150-500 × 10⁹/L
Serum creatinine	82	45-84 µmol/L
BUN	9.2	2.5-7.8 mmol/L
eGFR (CKD-EPI equation)	55	60-150 mL/min
Serum sodium (Na)	138	133-146 mmol/L
Serum potassium (K)	4.5	3.5-5.3 mmol/L
Serum calcium (Ca)	2.31	2.15-2.6 mmol/L
Serum phosphorus	1.03	0.8-1.5 mmol/L
CRP	4	0-5 mg/L
ALT	17	0-35 U/L
Bilirubin	9	0-21 µmol/L
HbA1c	38	0-41 mmol/mol
Iron	11	5.83-34.5 µmol/L
Transferrin	2.41	2-3.6 g/L
Transferrin saturation	18	15-50%
Ferritin	288	µg/L

She was previously diagnosed with toxic nodular goiter at 36 years of age after experiencing a 6 kg weight loss and significant adrenergic symptoms. Initial treatment consisted of carbimazole 15 mg three times daily, followed by partial thyroidectomy in the same year. Prior to her diagnosis of GD, she had not undergone TFTs for 20 years. Her significant past medical history included heart failure with diastolic dysfunction, AF, and hypertension, with no other autoimmune conditions diagnosed. She reported that her first cousin also has hyperthyroidism. Her regular medications included amlodipine, perindopril, apixaban, bumetanide, and simvastatin. On examination by her general practitioner (GP) at diagnosis, she was found to have an irregular heart rate but no tachycardia requiring beta-blockade. Other vital signs and systemic examinations were normal. Mild exophthalmos was noted, but no thyroid masses were palpable on neck examination.

**Figure 1:**
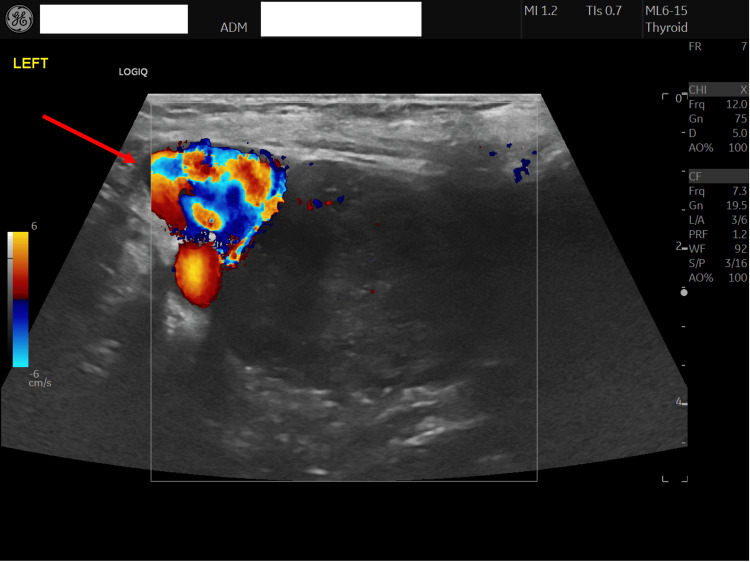
Thyroid ultrasound performed two months after the diagnosis of GD A 37 mm isoechoic nodule with peripheral blood flow is demonstrated in the left thyroid lobe (arrow), classified as U2. GD, Graves’ disease

The GP referred her to the district general hospital (DGH) endocrinology team, who advised starting carbimazole 15 mg once daily, with a plan to repeat TFTs every two months and titrate the dose accordingly. She was listed for an outpatient endocrinology review. The GP requested a thyroid ultrasound, which demonstrated a 37 mm benign isoechoic nodule in the left thyroid lobe, with a TI-RADS score of 1 (Figure 1). Repeat TFTs performed two months after starting carbimazole 15 mg showed a TSH of 0.011 mU/L, FT4 of 17.7 pmol/L, and free triiodothyronine (FT3) of 5.4 pmol/L (Table 2). The GP subsequently increased the carbimazole dose to 20 mg. Following this adjustment, no further TFTs were performed in primary care despite the endocrinology team’s prior recommendations, and no additional dose modifications were made.

**Table 2:** Results of TFTs performed since the diagnosis of GD, in relation to carbimazole or levothyroxine therapy FT3, free triiodothyronine; FT4, free thyroxine; GD, Graves’ disease; TFT, thyroid function test; TSH, thyroid-stimulating hormone

TSH (reference range: 0.27-4.2 mU/L)	FT4 (reference range: 11-22 pmol/L)	FT3 (reference range: 3.1-6.8 pmol/L)	Status of therapy at time of investigation	Change to the management plan following repeat TFTs
<0.005	39	No result available	At the diagnosis of GD, prior to the commencement of carbimazole	Started on carbimazole 15 mg once daily
0.011	17.7	5.4	Two months into therapy with carbimazole 15 mg once daily	Dose of carbimazole increased from 15 mg once daily to 20 mg once daily
38	3.5	<1.5	Five months into therapy with carbimazole 20 mg once daily	Carbimazole discontinued; commenced on levothyroxine 25 mcg once daily, uptitrated to 50 mcg once daily after three days
0.096	34.4	5.7	One month into therapy with levothyroxine 50 mcg once daily	Restarted on carbimazole 5 mg once daily
9.71	14	3	Two weeks into therapy with carbimazole 5 mg once daily	Carbimazole discontinued; restarted on levothyroxine 25 mcg once daily
0.195	31	2.4	One month into therapy with levothyroxine 25 mcg once daily	Levothyroxine discontinued
0.072	23.3	5.8	Not on any thyroid-related medications for one month	Repeat TFTs in two months and review ongoing therapy

**Figure 2:**
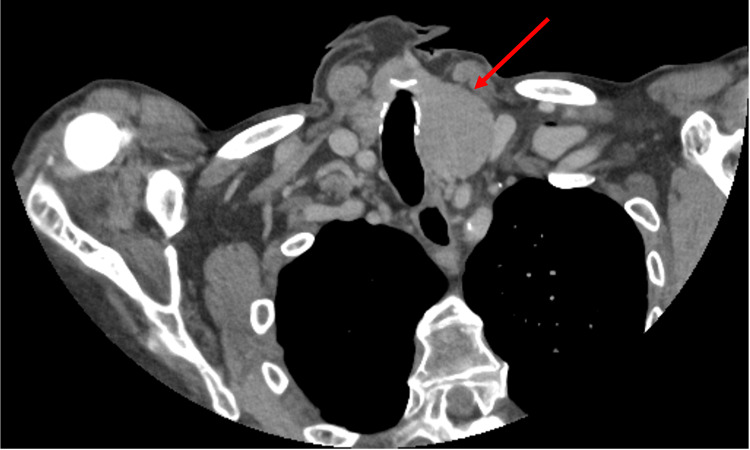
Contrast-enhanced CT scan of the neck performed five months after the diagnosis of GD An enlarged left thyroid lobe with contralateral tracheal deviation is demonstrated (arrow). GD, Graves’ disease

The patient continued to experience dysphagia and was reviewed by ENT. At this review, it was also noted that she had left-sided neck pain and hoarseness. A contrast-enhanced CT scan of the neck, requested by ENT, showed an enlarged left thyroid lobe measuring 3.6 × 3.6 × 4.7 cm with contralateral tracheal deviation (Figure 2). ENT planned that, if her symptoms did not improve within two months, referral for thyroidectomy would be considered.

**Figure 3:**
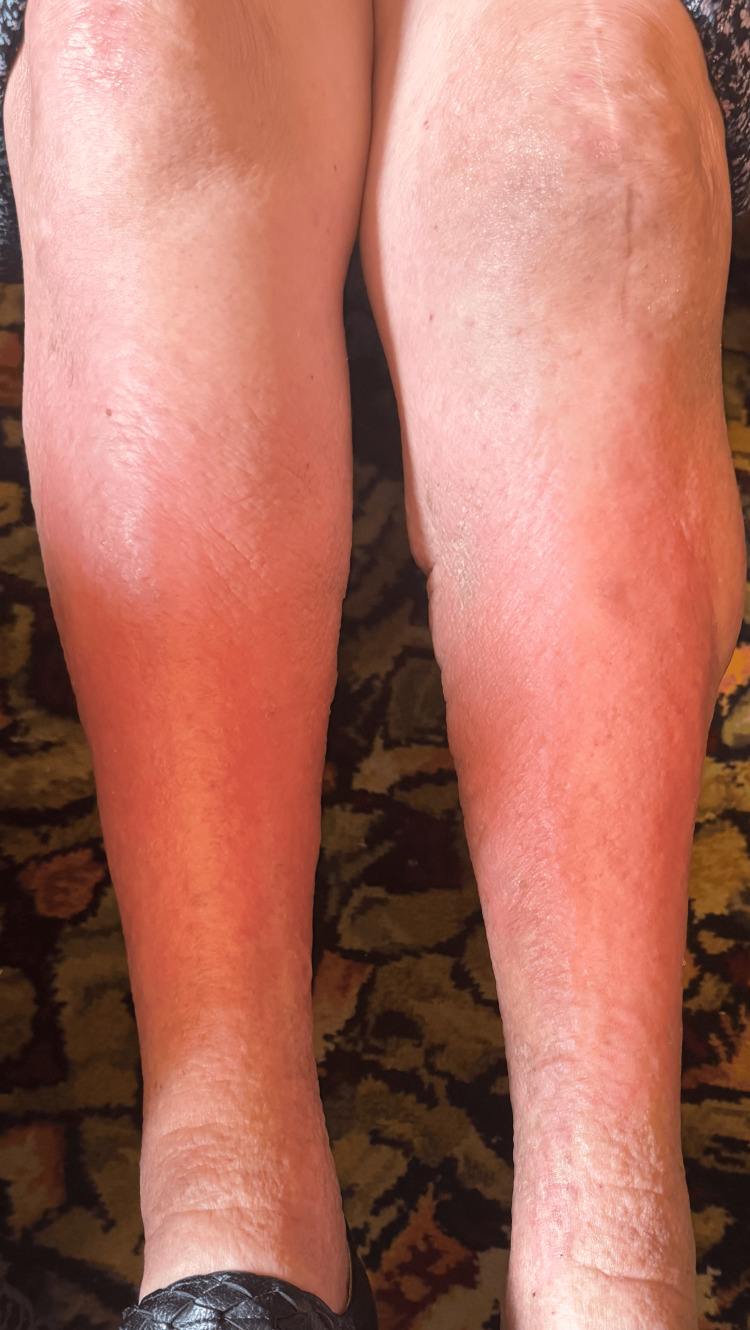
Photograph of the patient’s lower legs demonstrating bilateral pretibial myxedema

**Figure 4:**
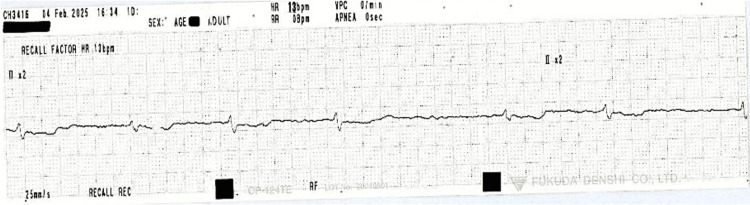
Telemetry strip showing AF with bradycardia, recorded during the patient’s first hospital admission for symptomatic hypothyroidism, thought to be secondary to carbimazole therapy Urgent TFTs at this time showed a TSH of 38 mU/L, FT4 of 3.5 pmol/L, and FT3 <1.5 pmol/L. AF, atrial fibrillation; FT3, free triiodothyronine; FT4, free thyroxine; TFT, thyroid function test; TSH, thyroid-stimulating hormone

The patient was admitted to the DGH seven months after her GD diagnosis with acute shortness of breath and lethargy. On examination, she had bilateral pretibial myxedema (Figure 3) and mild exophthalmos. An ECG showed AF with a heart rate of 36 bpm, which responded to atropine. She was also hypothermic with a temperature of 35.1 °C. Urgent repeat TFTs, performed seven months into treatment with carbimazole 20 mg once daily, showed a TSH of 38 mU/L, FT4 of 3.5 pmol/L, and FT3 <1.5 pmol/L. On endocrinology advice, carbimazole was discontinued, and levothyroxine 25 mcg once daily was initiated. This was uptitrated to 50 mcg prior to discharge, with plans for outpatient endocrinology follow-up in six weeks. During admission, the patient remained bradycardic, with her heart rate dropping to as low as 13 bpm (Figure 4).

The patient was readmitted one month later with a one-week history of redness, tightness, and swelling in both legs. She was treated for cellulitis and decompensated heart failure. An ECG again showed slow AF, but she remained asymptomatic with respect to bradycardia. TFTs during this admission showed a TSH of 0.096 mU/L and FT4 of 34.4 pmol/L, while she had been on levothyroxine for one month. On endocrinology advice, given her now hyperthyroid state, levothyroxine was discontinued, and carbimazole 5 mg once daily was restarted.

She was subsequently discharged but was readmitted only a week later with hypothermia, new confusion, decreased mobility, and persistent bradycardia. The endocrinology review noted exophthalmos. Repeat TFTs showed a TSH of 9.71 mU/L and FT4 of 14.0 pmol/L; carbimazole was therefore discontinued. Additional blood tests revealed normal cortisol, hyponatremia, and pancytopenia. Although not initially planned, levothyroxine 25 mcg once daily was restarted due to ongoing bradycardia.

A few days later, the patient was admitted to another DGH following an unwitnessed fall. She was bradycardic and clinically compromised, and a single-chamber permanent pacemaker was inserted in her best interests due to acute confusion. Urgent TFTs at this time showed a TSH of 0.195 mU/L, FT4 of 31 pmol/L, FT3 of 2.4 pmol/L, and TRAb level of 4.7 IU/L, while she had been on levothyroxine 25 mcg for one month. The endocrinology team advised discontinuing levothyroxine, with plans for outpatient follow-up. She was discharged a month later without any thyroid-directed medications. While alternating between carbimazole and levothyroxine therapy, the patient and her family consistently reported good compliance with treatment as prescribed.

After discharge, her GP repeated TFTs one week later, at which point she had not been on any thyroid-related medication for a month. Results showed a TSH of 0.072 mU/L, FT4 of 23.3 pmol/L, and T3 of 5.8 pmol/L. Thyroid function will be reassessed in two months, with further outpatient endocrinology review to determine an ongoing management plan. Given the patient’s frailty and comorbidities, thyroidectomy has not been considered due to the high risk of complications, and a block-and-replace regimen was also deemed inappropriate. As the patient has a degree of Graves’ orbitopathy, this may also influence her suitability for RAI in the future.

## Discussion

GD is an autoimmune condition defined by the presence of IgG autoantibodies against the TSH-R. These autoantibodies (TRAbs) activate the TSH-R, leading to uncontrolled synthesis and secretion of the thyroid hormones thyroxine (T4) and triiodothyronine (T3) from follicular cells [4]. Symptoms include heat intolerance, tremor, diarrhea, muscle weakness, palpitations, and unintentional weight loss. In addition, patients with GD may experience extrathyroidal manifestations, including Graves’ orbitopathy, which occurs in approximately 25% of cases [5], as well as thyroid acropachy and pretibial myxedema. A rare complication of undiagnosed or untreated GD is thyroid storm, in which patients can present with fever, tachycardia, delirium, and coma [6].

A diagnosis of GD is confirmed biochemically by elevated TRAbs, suppressed TSH, and increased circulating thyroid hormones. ATDs, such as carbimazole or propylthiouracil [4], remain one of the mainstays of treatment. A beta-blocker such as propranolol may also be prescribed to improve adrenergic symptoms if appropriate. Due to the low remission rate associated with GD and the inability to treat toxic nodular goiter with ATDs alone, RAI is increasingly used as a first-line treatment [7]. However, RAI is contraindicated in patients with active Graves’ orbitopathy due to the risk of exacerbation. Thyroidectomy is another therapeutic option in selected cases.

The clinical manifestations of hyperthyroidism in elderly patients may differ from those observed in younger individuals. Symptoms are often more subtle or attributed to other age-related conditions, making recognition of hyperthyroidism more challenging [8]. Instead of presenting with adrenergic symptoms, elderly patients may present with weight loss, AF, and congestive heart failure. Cardiac complications are particularly prevalent in patients over 60 years of age with preexisting heart disease [9]. Patients over 60 with a chronically suppressed TSH are three times more likely to develop AF in the following decade [8], and AF with a slow ventricular rate is observed in up to 20% of hyperthyroid patients [10]. In addition, GD is associated with cardiomyopathy, cardiac valve involvement, and pulmonary arterial hypertension [9]. An alternative presentation of hyperthyroidism in the elderly is neurocognitive change, including fatigue, depression, cognitive decline, anorexia, and apathy, a constellation termed “apathetic thyrotoxicosis” [11]. A cross-sectional study by Boelaert et al. proposed that in patients over 60, there should be a lower threshold for performing TFTs, particularly in those presenting with AF and weight loss [12]. The initial management of elderly patients with severe thyrotoxicosis should mirror that of younger patients, with a course of ATD to achieve euthyroidism, alongside beta-blockers if required [4]. However, according to the 2018 ETA guideline for the management of GD, older patients who present with AF or other cardiac compromise should be considered for early definitive therapy, usually RAI, to reduce the risk of further cardiac complications from recurrent hyperthyroidism [4].

Although the diagnoses of AF and heart failure predated this patient’s diagnosis of GD, due to the lack of TFTs performed between 2004 and 2024, it is uncertain how long she had been in a hyperthyroid state. Thus, it remains unclear whether both cardiac manifestations can be directly attributed to hyperthyroidism, although they were undoubtedly exacerbated by it. A transthoracic echocardiogram (Table 3) performed during one of the patient’s hospital admissions showed diastolic dysfunction with biatrial dilation, a high probability of pulmonary hypertension, and regurgitation of the aortic, mitral, and tricuspid valves. Her bradycardia was attributed to carbimazole-induced hypothyroidism that did not resolve despite attempts to correct thyroid status, thought to be secondary to advanced age and preexisting heart failure with subsequent hypervolemic hyponatremia.

**Table 3:** Conclusions of imaging studies performed following the diagnosis of GD, both as an outpatient and during multiple hospital admissions AF, atrial fibrillation; EF, ejection fraction; GD, Graves’ disease; LV, left ventricle; LVH, left ventricular hypertrophy; PG, pressure gradient; TR, tricuspid regurgitation; U2, ultrasound classification 2 (benign)

Study type	Finding(s)
CT chest, abdomen, and pelvis	Enlarged left thyroid lobe with no suspicious features identified. Evidence of liver cirrhosis and heart failure. No evidence of malignancy.
Ultrasound scan of the thyroid gland	Small amount of residual thyroid tissue (query isoechoic nodule) in the right lobe with no concerning features. The left lobe contains a 37 mm isoechoic nodule demonstrating marked peripheral blood flow. No tracheal deviation or retrosternal extension. Classified as U2 (benign). The right lobe is difficult to interpret.
CT neck with contrast	Enlarged left thyroid lobe measuring 3.6 × 3.6 × 4.7 cm, causing contralateral tracheal deviation without significant narrowing. No significant retrosternal extension.
Trans-thoracic echocardiography	Left ventricle normal in size with concentric LVH by LV geometry. Borderline low systolic function with a visual EF estimate of 50-55%. Evidence of diastolic dysfunction. Mildly dilated right ventricle with impaired systolic function. Bi-atrial dilatation. Mild aortic regurgitation, mitral prolapse with regurgitation, and mild-to-moderate tricuspid regurgitation. TR max PG: 54.5 mmHg, indicating high probability of pulmonary hypertension. Pleural effusion noted.
24-hour Holter ECG	Baseline rhythm: AF with frequent episodes of bradycardia (in the context of AF). Total duration: 12 hours 34 minutes (burden of 51.7%), with the longest episode lasting 477 beats at 23 bpm (one-minute HR = 30 bpm). Slowest episode: 21 bpm lasting 187 beats (one-minute HR = 31 bpm). Very frequent pauses observed in the context of slow AF (maximum duration: 2.93 seconds). Episodes of bradycardia included the longest (477 beats) and slowest (21 bpm) episodes. Occasional multifocal ventricular ectopics seen in isolation, couplets, and short runs of bigeminy (maximum of four cycles). Mean HR: 41 bpm; max HR: 74 bpm; min HR: 28 bpm. Conclusion: Pauses noted in the context of slow AF, with rates ranging from 21 to 41 bpm.

Following the diagnosis of GD, she was initially started on carbimazole 15 mg daily, an appropriate dose according to current prescribing guidance. Two months later, her GP increased the dose to 20 mg following repeat TFTs requested by the endocrinology team. These showed an improvement in TSH from <0.005 mU/L to 0.011 mU/L and in FT4 from 39 pmol/L to 17.7 pmol/L. Given that thyroid function had already improved on 15 mg carbimazole, it could be argued that increasing the dose to 20 mg without endocrinology input was inappropriate.

According to NICE guidance, when titrating carbimazole, TFTs should be monitored every six weeks until TSH normalizes, and then TSH with cascading should be checked every three months until ATD discontinuation. After discontinuation, TSH with cascading should be checked within eight weeks, then every three months for a year, and annually thereafter [13]. However, after her carbimazole dose was increased from 15 mg to 20 mg, no further TFTs were performed until her hospital admission four months later. Although the reasons for this gap in monitoring remain unclear, it may reflect challenges faced in primary care regarding laboratory follow-up for thyroid disease. Furthermore, the GP did not inform the endocrinology team of the dose adjustment; as a result, the endocrinologists were unaware of the change and did not request repeat TFTs. Timely monitoring of TFTs is essential to reduce the risk of both iatrogenic hypothyroidism and hyperthyroidism, particularly in elderly patients who may be more sensitive to fluctuations in thyroid function.

When a patient’s thyroid status alternates between over- and underactivity, particularly with low doses of levothyroxine, this raises the possibility of oscillating GD. Oscillating GD is a rare phenomenon in which both TSH-R-stimulating and TSH-R-blocking antibodies coexist, and the balance between these determines whether the patient is clinically hyperthyroid or hypothyroid. Most published case reports describe this switch occurring several years after cessation of ATDs [14]. However, our patient first became hypothyroid during her initial hospital admission, only six months after starting carbimazole. Given the rarity of oscillating GD, it was thought more likely that her fluctuations reflected increased sensitivity to thyroid suppression or replacement therapy due to advanced age. At diagnosis, her TRAb level was 9.7 IU/L, and seven months later, it was 4.7 IU/L; thus, it was measured only twice. According to the 2018 European Thyroid Association guideline for GD, TRAb levels should be reassessed after 12-18 months of carbimazole therapy to determine the need for ongoing treatment [4]. Therefore, in this case, additional TRAb monitoring was not considered necessary.

## Conclusions

The clinical manifestation of GD in elderly patients often differs from that seen in younger individuals, making recognition and diagnosis more challenging. In this population, untreated hyperthyroidism can present with cardiac complications such as AF and heart failure, while GD specifically carries an additional risk of valvular involvement and pulmonary arterial hypertension. This patient’s presentation without classic adrenergic features of thyrotoxicosis highlights the importance of considering thyroid dysfunction as a differential in older patients presenting with arrhythmias or heart failure, where timely treatment may help prevent further complications.

Management in the elderly can be further complicated by limited therapeutic options and the heightened sensitivity to small fluctuations in ATD or levothyroxine dosing. In this case, both thyroid dysfunction and repeated hospital admissions adversely affected the patient’s functional and neurocognitive baseline, underscoring the importance of careful recognition, close monitoring, and tailored management of GD in older adults.
